# Predictors of incident herpes simplex virus type 2 infections in young women at risk for unintended pregnancy in San Francisco

**DOI:** 10.1186/1471-2334-7-113

**Published:** 2007-09-26

**Authors:** Nicholas J Moss, Cynthia C Harper, Katherine Ahrens, Katherine Scott, Susan Kao, Nancy Padian, Tina Raine, Jeffrey D Klausner

**Affiliations:** 1 STD Prevention and Control Services; Dept. Public Health, San Francisco, California, USA; 2 Bixby Center for Reproductive Health Research and Policy; Dept. Obstetrics, Gynecology and Reproductive Sciences, University of California, San Francisco, USA; 3 Department of Medicine, University of California, San Francisco, USA

## Abstract

**Background:**

Young women receiving family planning services are at risk for both unintended pregnancy and herpes simplex virus type 2 (HSV-2) infection.

**Methods:**

We performed a secondary analysis using data from a previously published randomized controlled trial evaluating access to emergency contraception on reproductive health outcomes. Women aged 15 to 24 years were recruited from two Planned Parenthood clinics and two community health clinics in San Francisco. Demographic information and sexual history were obtained by interview. HSV-2 seropositivity was determined by fingerstick blood test. New pregnancies were measured by self-report, urine testing and medical chart review. Subjects were evaluated for incident HSV-2 infection and pregnancy at a 6-month follow-up appointment. Women who were pregnant or intending to become pregnant at enrolment were excluded.

**Results:**

At enrolment 2,104 women were screened for HSV-2 and 170 (8.1%) were seropositive. Eighty-seven percent of initially seronegative women completed the study (n = 1,672) and 73 (4.4%) became HSV-2 seropositive. HSV-2 seroincidence was 7.8 cases per 100 person-years. One hundred and seventeen women (7%) became pregnant and 7 (6%) of these had a seroincident HSV-2 infection during the study. After adjustment for confounders, predictors of incident HSV-2 infection were African American race and having multiple partners in the last six months. Condom use at last sexual encounter was protective.

**Conclusion:**

HSV-2 seroincidence and the unintended pregnancy rate in young women were high. Providers who counsel women on contraceptive services and sexually transmitted infection prevention could play an expanded role in counselling women about HSV-2 prevention given the potential sequelae in pregnancy. The potential benefit of targeted screening and future vaccination against HSV-2 needs to be assessed in this population.

## Background

Sexually active young women in the United States are exposed to herpes simplex virus type 2 (HSV-2) with high frequency. There currently is no cure for HSV-2 and clinical manifestations may be controlled with periodic or long-term suppressive medical therapy. Few (10%) infected individuals report symptomatic disease, typically consisting of painful genital sores, although forward transmission is possible with no reported symptoms. In addition, HSV-2 seropositivity is a known risk factor for human immunodeficiency virus (HIV) acquisition [1,2].

HSV-2 infections are known to have especially serious clinical sequelae in neonates. In general, women with pre-pregnancy HSV-2 seropositivity have a reduced risk of neonatal transmission, attributed to the passage of transplacental antibodies to the foetus, but women with a recurrent herpes episode at delivery generally undergo caesarean section to prevent transmission to the neonate [3-5]. Pre-pregnancy seronegative women with an incident herpes infection are also at high risk for HSV-2 transmission and neonatal complications [6].

National Health and Nutrition Examination Survey (NHANES) surveillance data collected from 1999 through 2004 found an HSV-2 seroprevalence of 23.1% in US females aged 14 to 49 years [7]. While overall HSV-2 seroprevalence has declined in recent years and is estimated to be only 2.3% in females aged 14 to 19 years, it increases to 15.6% in females aged 20 to 29 years indicating high acquisition rates during this age transition [7].

In San Francisco, young women under age 25 account for the highest heterosexually transmitted incidence rates of reportable sexually transmitted diseases (STD), such as *Chlamydia trachomatis *(CT) and *Neisseria gonorrhea *(GC) [8]. This population is at high risk of HSV-2 acquisition, although new infections are not reportable making incidence measurements challenging. These young women are most likely also at high risk for unintended pregnancy. In national data from 2001, high risk of unintended pregnancy was associated with age 18 to 24 years, low-income, minority racial status, and failure to complete high school [9]. The risk of HSV-2 infection in women with high unintended pregnancy rates presents a particularly concerning situation.

With this study we sought to determine predictors of incident HSV-2 infection and concurrent unintended pregnancy in a racially diverse cohort of young, sexually active urban women seeking family planning services.

## Methods

Between July 2001 and June 2003 women between the ages of 15 and 24 years were recruited from four clinics serving adolescents and young adults with family planning needs in San Francisco City and County, California. The resulting cohort was assembled as part of a randomized controlled trial (RCT) evaluating access to emergency contraception on reproductive health outcomes. A complete description of the methods has been published elsewhere [10]. In brief, the clinics included two Planned Parenthood clinics, a college health centre providing primary care services, and a community clinic for young adults. Women attending scheduled appointments or unscheduled drop-in visits as well as those women accompanying them were referred to study personnel. English and Spanish speaking women were then screened for eligibility to participate. Women in the targeted age range who reported sexual intercourse in the previous six months were eligible for the study. Pregnant women, women refusing a pregnancy test, women wishing to become pregnant in the next six months and women using low compliance contraceptive methods – injectables, intrauterine devices (IUDs), or implants – were excluded. Women who reported unprotected intercourse in the previous three days were also excluded. These exclusions were necessary for the purposes of the RCT emergency contraception study. Reason for study exclusion only was collected on women ineligible for study participation.

Study personnel interviewed all participants at enrolment using a standardized questionnaire collecting demographic information, sexual history and risk behaviours. All information was self-reported and not verified by review of medical records.

Participants were then tested for HSV-2 antibodies by POCKit HSV-2 rapid point-of-care test (Diagnology Inc, Belfast, Northern Ireland) on fingerstick whole blood samples. POCKit tests were donated by the manufacturer for study use and the test was considered less invasive than venipuncture and thus more acceptable for study participants. This test is now available as "*biokit*HSV-2 Rapid Test" from Biokit USA, Lexington, MA or as "SureVue-HSV-2" Rapid Test from Fisher HealthCare, Houston, TX. Premarket evaluation of this test against culture-documented and Western blot confirmed HSV-2 negative (n = 50) and HSV-2 positive (n = 253) showed POCKit HSV-2 test sensitivity was 96% and specificity was 98% [11]. Sensitivity for HSV-2 was not affected by the presence of HSV-1 antibodies. In this premarket evaluation, discordant specimens were further examined by repeat Western blot testing. POCKit falsely identified one specimen as positive for HSV-2 (unknown reason for false positive) and falsely identified eight specimens as negative for HSV-2 (due to low titre levels in the specimens).

Participants also submitted a urine specimen for pregnancy testing (Clearview One Step, Unipath Diagnostics, Waltham, MA, or equivalent) and *Chlamydia trachomatis *(CT) nucleic acid amplification testing (BD ProbeTec, Becton Dickinson & Co, Sparks, MD).

Each participant had a follow-up interview approximately six months later at either the enrolling clinic or at her home. Interviews were conducted by telephone when necessary; however, women who had telephone follow-up were unavailable for repeat HSV-2 and pregnancy testing. This second interview asked participants about any new STI diagnosis, pregnancy or change in risk behaviours since enrolment using a standardized questionnaire. Repeat HSV-2 and pregnancy testing, as well as a medical chart review for interim HSV-2 infection or pregnancy, were also performed at the 6-month follow-up, when possible.

All subjects were compensated with $10 at enrolment and $20 at follow-up. All women who tested positive for CT at enrolment were referred to a clinic for treatment and partner management per clinic protocol. Women testing positive for HSV-2 antibodies were only informed and counselled on HSV-2 antibody results if the individual participant so requested, as routine HSV-2 screening of patients was not the standard of care at participating clinics. Women with positive rapid HSV-2 antibody results were told they could seek confirmatory testing at their discretion, however, confirmatory test results were not recorded as a part of this study. Information on number and characteristics of participants requesting HSV-2 results was not recorded. Pregnancy outcomes of participants with HSV-2 infection were also not recorded, as study participation was complete at six months.

The primary outcome assessed was HSV-2 seroconversion during the study period. The secondary outcome was incident pregnancy among those with follow-up testing for HSV-2.

### Statistical Analysis

Approximately twenty baseline demographic, historical, biological and behavioural measures that, based on relevant literature, might be associated with HSV-2 were individually evaluated for significance for both prevalent and incident HSV-2 infection. Dichotomous variables were evaluated for significance with the Chi-squared test; multilevel categorical variables were analyzed using a General Chi-squared test. Odds ratios (OR) and 95% confidence intervals (CI) were generated with logistic regression for incident HSV-2 infection among select variables. Factors previously reported to be predictors of HSV-2 as well as factors approaching significance (p ≤ 0.1) in the bivariate logistic regression analysis were included in an initial multivariable model. The final model was constructed using a combination of automatic and manual reverse stepwise selection. Frequency tables and statistical computations were performed using Stata 9.1 (Stata Corporation, College Station, TX) and SAS 9.1 (SAS Institute Inc., Cary, NC). Incidence densities were calculated by dividing incident events by the total time between enrolment and either follow-up appointment or time of incident event for all participants. Incident HSV-2 infection or pregnancy was assumed to have occurred mid-way through the follow-up interval. Ninety-five percent exact confidence limits for incidence density estimates were calculated using SAS.

Informed consent for participation in the study was obtained from all women. The University of California San Francisco (UCSF) Committee on Human Research and Planned Parenthood approved the study, including all methods, questionnaires and consent documents.

## Results

In total, 4,361 women from the four clinics were screened for study eligibility. Fifty-one percent did not meet RCT emergency contraception study eligibility requirements with 15% reporting unprotected intercourse in the previous 3 days, 10% using injectable contraceptives, 9% not receiving a pregnancy test, 7% not sexually active in last 6 months, 2% pregnant, and 8% ineligible for other reasons. Of the 2,130 women eligible for the emergency contraception study, 13 refused participation, 13 did not have HSV-2 testing at baseline, and 2,104 were enrolled in the HSV-2 study. At enrolment, 170 women (8.1%) tested positive for HSV-2 serum antibodies. HSV-2 seropositivity by selected demographic and behavioural characteristics at enrolment is presented in Table 1.

**Table 1:** HSV-2 seropositivity by select demographic and behavioural factors at baseline

Risk Factors		Total (%)	HSV-2 seropositivity		P value
				
			n	%	
Total		2104 (100)	170	8.1	

Race					

	White	647 (30.8)	42	6.5	<0.0001
	Latina	419 (19.9)	43	10.3	
	Black	312 (14.8)	42	13.5	
	API	467 (22.2)	20	4.3	
	Multi/Other	259 (12.3)	23	8.9	

Age					

	15–19	959 (45.6)	52	5.4	<0.0001
	20–24	1145 (54.4)	118	10.3	

Age at first sexual intercourse					

	>15 years	1214 (57.7)	84	6.9	0.023
	≤ 15 years	890 (42.3)	86	9.7	

Ever pregnant					

	No	1425 (67.7)	90	6.3	< 0.0001
	Yes	679 (32.3)	80	11.8	

Ever diagnosed with an STI (N = 2101)					

	No	1621 (78.5)	102	6.3	< 0.0001
	Yes	445 (21.5)	68	15.3	

Number of partners in last 6 months					

	1 partner	1514 (72.0)	114	7.5	0.138
	≥ 2 partners	590 (28.0)	56	9.5	

Reason for visit					

	OCPs	516 (24.5)	41	8.0	0.100
	Pregnancy Test	125 (5.9)	8	6.4	
	STI/HIV Test	250 (11.9)	31	12.4	
	Annual/Pap	496 (23.6)	40	8.1	
	Other^a^	710 (33.7)	50	6.8	

HSV-2, herpes simplex virus type 2; API, Asian or Pacific Islander; OCP, oral contraceptive pill; STI, sexually transmitted infection; HIV, Human Immunodeficiency Virus; Pap, Papinikolaou smear.

^a ^Other reasons for visit included: colposcopy, miscellaneous reproductive health needs, other health complaints, no reason given, or no appointment at enrolment.

Of the 1,934 women who were HSV-2 seronegative at enrolment, 1,672 (86.5%) had repeat HSV-2 testing performed at six months. The remaining 262 women were lost to follow-up or did not have HSV-2 lab results available from follow-up testing. The mean time to follow-up was 6.9 +/- 1.3 months. Seventy-three (4.4%) of those women tested at follow-up were found to be seropositive for HSV-2 antibodies, which is equivalent to an incidence rate of 7.8 (3.3 – 15.5) cases per 100 person-years.

**Figure 1:**
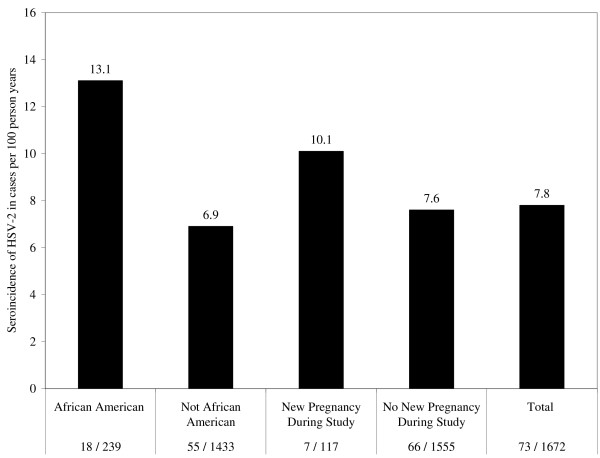
Herpes simplex virus type 2 (HSV-2) seroincidence in women from San Francisco aged 15 to 24 years by African American race and new pregnancy during the 6-month study period. Seroincidence expressed as cases per 100 person years. N = 1672.

Of the 1,672 women with HSV-2 laboratory follow-up, 117 (7.0%) became pregnant during the study, resulting in an incidence rate of 12.7 (6.7 – 21.9) unintended pregnancies per 100 person-years. Seven women (0.4%) had both incident HSV-2 infection and pregnancy during the study. Among the 117 women who became pregnant during the study period, the incidence rate of HSV-2 seroconversion was 10.1 (4.9 – 18.5) cases per 100 person-years. The temporal relationship between HSV-2 infection and date of conception was not defined by our data. HSV-2 incidence by African American race and unintended pregnancy during the study is presented in Figure 1.

In the bivariate analysis several factors, all assessed at enrolment, were significantly associated with an increased risk of incident HSV-2 infection including African American race (OR 2.14 [1.10–4.16]), history of pregnancy (OR 1.80 [1.12–2.89]), CT infection at enrolment (OR 2.62 [1.15–5.95]), multiple sex partners within the last six months (OR 1.63 [1.01–2.65]), last sexual intercourse was unprotected (OR 2.27 [1.22–4.25]), last partner over 25 years old (OR 1.70 [1.04–2.77]), and pressured into sex in last six months (OR 2.21 [1.03–4.76]). Condom use at last intercourse was found to be protective (OR 0.62 [0.39–1.00]). After adjusting for confounders in multivariable regression analysis, however, only African American race (OR 2.31 [1.28–4.14]), multiple sex partners within the last six months (OR 1.75  [1.07–2.89]) and the protective effect of condom use at last intercourse remained significant (OR 0.56 [0.34–0.92]). These results are summarized in Table 2.

**Table 2:** HSV-2 seroconversion by selected demographic and behavioural factors

	HSV-2 seroconversion		COR (95% CI)	AOR (95% CI)
	%	n/N		
Total	4.4	73/1672		

Race				

White	3.7	19/519	Referent	
Latina	3.7	12/322	1.02 (0.49–2.13)	
Black	7.5	18/239	2.14 (1.10–4.16)	2.31 (1.28–4.14)
API	3.6	14/389	0.98 (0.49–1.99)	
Multi/Other	4.9	10/203	1.36 (0.62–2.99)	

Age				

15–19	3.6	29/801	Referent	
20–24	5.1	44/871	1.42 (0.88–2.29)	0.89 (0.50–1.58)

Age at first sexual intercourse				

> 15 years	4.7	45/960	Referent	
≤ 15 years	3.9	28/712	0.83 (0.51–1.35)	

Ever pregnant				

No	3.6	41/1156	Referent	
Yes	6.2	32/516	1.80 (1.12–2.89)	1.61 (0.99–2.63)

Ever diagnosed with an STI (N = 1640)				

No	4.0	53/1312	Referent	
Yes	6.1	20/328	1.54 (0.91–2.62)	

CT-infected at enrollment (N = 1640)				

No	4.2	66/1572	Referent	
Yes	10.3	7/68	2.62 (1.15–5.95)	

Number of partners in last 6 months				

1 partner	3.7	45/1203	Referent	
≥ 2 partners	6.0	28/469	1.63 (1.01–2.65)	1.75 (1.07–2.89)

Any unprotected sexual intercourse in last 6 months (N = 1670)				

No	3.6	32/900	Referent	
Yes	5.3	41/770	1.53 (0.95–2.45)	

Last sexual intercourse was unprotected (N = 1671)				

No	4.0	60/1519	Referent	
Yes	8.6	13/152	2.27 (1.22–4.25)	

Currently uses condoms (N = 1671)				

No	4.9	29/592	Referent	
Yes	4.1	44/1079	0.83 (0.51–1.33)	

Condom use at last intercourse (N = 1671)				

No	5.5	40/728	Referent	
Yes	3.5	33/943	0.62 (0.39–1.00)	0.56 (0.34–0.92)

Pressured into having sex in last 6 months (N = 1667)				

No	4.1	65/1575	Referent	
Yes	8.7	8/92	2.21 (1.03–4.76)	

Last sexual partner over 25 years old (N = 1672)				

No	3.7	46/1235	Referent	
Yes	6.2	27/437	1.70 (1.04–2.77)	1.58 (0.90–2.76)

HSV-2, herpes simplex virus type 2; COR, crude odds ratio; AOR, adjusted odds ratio; CI, confidence interval; API, Asian or Pacific Islander; STI, sexually transmitted infection; CT, *Chlamydia trachomatis*.

## Discussion

HSV-2 seroincidence and the unintended pregnancy rate in young women receiving family planning services in San Francisco clinics over a 6-month period were high at 7.8 and 12.7 per 100 person-years, respectively. The rate of HSV-2 acquisition in women who became pregnant during the study period was 10.1 cases per 100 person-years. Seroincidence of HSV-2 has recently been reported as 7.3 cases per 100 person-years in a study of 100 sexually active females aged 14 to 17 years [12]. A larger study found a HSV-2 seroincidence of 13.2 cases per 100 person-years in males and females aged 14 to 24 at inner city STD clinics in the mid 1990's [13]. Our own data, along with these, are consistent with the increase in HSV-2 seroprevalence observed nationally as women move from adolescence into their late twenties [7]. We are unaware of other studies demonstrating high rates of unintended pregnancy in a population of young women that also has high seroincident HSV-2.

Recent NHANES data showed higher HSV-2 seroprevalence associated with increasing age, younger age at first intercourse, number of lifetime sexual partners, and poverty [7]. African American women also had relatively high HSV-2 seropositivity [7]. While bivariate analysis of our data suggested several baseline behavioural and demographic predictors of HSV-2 seroincidence, multivariable analysis found African American race and multiple sex partners within the last six months increased the likelihood of new infection. The risks were high: African American women were nearly 2.5 times more likely to acquire HSV-2 infection than non-African American women and those with multiple partners were 75% more likely. Clinicians should be aware of the significant increased risk associated with these factors.

Our findings showed condom use at the last intercourse prior to enrolment was highly protective, associated with a 44% reduction in likelihood for acquiring HSV-2 after adjustment for confounders. This is consistent with a recent study by Wald et al showing that STD clinic patients reduced their risk for HSV-2 by 26% as they increased condom use frequency [14]. Our study is at least the fourth to demonstrate that condom use was associated with decreased HSV acquisition and should encourage medical providers and policy makers to feel confident in recommending condom use to decrease HSV-2 transmission [13-15].

We observed a high rate of unintended pregnancies in an urban, racially diverse group of young women. This concurs with findings from the National Survey of Family Growth for 2001 that showed poor, uneducated, minority women are at increased risk for unintended pregnancy [9]. Interestingly, nearly half of these unintended pregnancies occurred in women reporting concurrent contraceptive use [9]. While participants did not intend to become pregnant in the next six months at enrolment, they could have changed their minds after enrolment and intentionally conceived before the 6-month follow up appointment. Information regarding change in intention in the interim was not collected.

The role of HSV in the development of severe neonatal complications following maternal infection is well known [3-5]. Brown et al recently reported a neonatal herpes infection rate of 1 in 3200 live births (31.25 per 100,000 live births) for several hospitals around Seattle, Washington [6]. Others have reported incidences of 11 to 33 infections per 100,000 live births [16-18]. Rates of neonatal herpes are highest in neonates born to recently HSV-infected women who were seronegative pre-pregnancy [6]. Our data suggest that young women in San Francisco run a high risk of acquiring HSV-2 infection shortly before and during their pregnancies.

These findings highlight the need for multiple interventions to minimize acquisition of HSV-2 infection in the young urban female population. Possible effective interventions include targeted screening and vaccination among women seeking family planning services as these women are at high risk for unintended pregnancy, despite contraceptive use. Targeted screening could include women who are African American or who report multiple sex partners within the last six months. We think it is also important for providers who counsel women on contraceptive services and STI prevention to play an expanded role in counselling women about prevention of HSV-2 infection given the potential sequelae in pregnancy. While vaccine development requires extensive bench to bedside translational research, the other interventions listed are well within the grasp of existing public health institutions and future studies should evaluate their effectiveness in reducing the burden of new HSV-2 infections in young women at risk for unintended pregnancy.

Also pertinent to this discussion, it has been suggested that HSV-2 antibody screening may place unnecessary psychological stress on individuals, as there is typically no intervention indicated for asymptomatic cases [19,20]. Ample research by our group and others, however, indicates that HSV-2 testing does not cause inappropriate levels of psychological distress and thus prevention strategies that incorporate routine HSV-2 testing may be warranted [21-24]. At the least, the evaluation of such strategies should be a public health priority.

With regard to limitations, the main study, of which this is a sub-study, was designed to examine emergency contraception on reproductive health outcomes in women using high compliance contraceptives not intending to become pregnant in the next six months. These criteria resulted in the exclusion of half of the women initially screened. Women using methods requiring a lower degree of user compliance, including injectable contraception and IUDs, would be expected to have a lower unintended pregnancy rate. Because women using low compliance methods were excluded, the unintended pregnancy rate calculated in our study may be an overestimate of the actual rate. However, these women tend to use condoms less frequently and, as a result, their exclusion may have led to an underestimation of HSV-2 seroincidence in our study. A cohort assembled with incident HSV-2 infection and pregnancy as the sole primary research endpoints might have avoided these potential biases.

Fourteen percent of women did not complete HSV-2 testing at 6-month follow-up. While the majority (64%) of these women were lost to follow-up, those remaining completed telephone interviews at follow-up for the main study but were not available for repeat fingerstick blood testing for HSV-2 or pregnancy testing. It is unknown how this impacted the measures of incidence or of associations. An analysis of attrition for the main study showed no significant differences in baseline demographic traits, history of pregnancy or STIs, or contraceptive methods between those who completed the study and those lost to follow-up.

In addition, we relied on the self-report of sexual risk behaviours, which women may recall with varying degrees of accuracy. This is a limitation of our study and, indeed, of other studies seeking to characterize behavioural factors affecting the acquisition of sexually transmitted infections. By contrast, the use of both urine testing, self-report and medical chart review in our assessment of new pregnancies is a strength of our study, although it is possible that some women may have under-reported new pregnancies during the study period that were terminated before urine testing at follow-up.

Finally, although the rapid point-of-care test we used to identify HSV-2 infection has a documented high sensitivity (96%) and specificity (98%), it is not as accurate as Western blot. A few women may have been falsely identified as HSV-2 positive while others may have been falsely identified as HSV-2 negative, especially if they had low HSV-2 titre levels. This would result in non-directional misclassification and would bias our findings towards not finding significant predictors of HSV-2 infection. Therefore our findings may have been stronger if we had used a more accurate test.

## Conclusion

To conclude, our prospective cohort study of young, sexually active women characterized the epidemiology of incident HSV-2 infection in this important population. The inclusion of incident pregnancy data demonstrates the considerable ongoing threat of new HSV-2 infection in women who are pregnant or at risk of unintended pregnancy. Clinicians need to have a clear idea of the risk of exposure to HSV-2 in young women who have a high likelihood of becoming pregnant regardless of pregnancy intentions. Specific HSV-2 education should be routinely offered to women obtaining family planning services.

## Competing interests

The author(s) declare that they have no competing interests.

## Authors' contributions

NJM performed the analysis and drafted the manuscript; CCH designed and implemented the study; KA performed the analysis and revised the manuscript; KS performed initial analysis and revised the manuscript; SK performed initial analysis and revised the manuscript; NP designed and implemented the study and revised the manuscript; TR designed and implemented the study and revised the manuscript; JDK designed and implemented the study and revised the manuscript. All authors read and approved the final manuscript.

## Pre-publication history

The pre-publication history for this paper can be accessed here:


